# Discovery Analysis of TCGA Data Reveals Association between Germline Genotype and Survival in Ovarian Cancer Patients

**DOI:** 10.1371/journal.pone.0055037

**Published:** 2013-03-21

**Authors:** Rosemary Braun, Richard Finney, Chunhua Yan, Qing-Rong Chen, Ying Hu, Michael Edmonson, Daoud Meerzaman, Kenneth Buetow

**Affiliations:** 1 Robert H. Lurie Comprehensive Cancer Center, Northwestern University, Chicago, Illinois, United States of America; 2 Center for Biomedical Informatics and Information Technology, National Cancer Institute, National Institutes of Health, Bethesda, Maryland, United States of America; 3 Computational Science and Informatics Program, Complex Adaptive Systems Initiative, Arizona State University, Phoenix, Arizona, United States of America; Ohio State University Medical Center, United States of America

## Abstract

**Background:**

Ovarian cancer remains a significant public health burden, with the highest mortality rate of all the gynecological cancers. This is attributable to the late stage at which the majority of ovarian cancers are diagnosed, coupled with the low and variable response of advanced tumors to standard chemotherapies. To date, clinically useful predictors of treatment response remain lacking. Identifying the genetic determinants of ovarian cancer survival and treatment response is crucial to the development of prognostic biomarkers and personalized therapies that may improve outcomes for the late-stage patients who comprise the majority of cases.

**Methods:**

To identify constitutional genetic variations contributing to ovarian cancer mortality, we systematically investigated associations between germline polymorphisms and ovarian cancer survival using data from The Cancer Genome Atlas Project (TCGA). Using stage-stratified Cox proportional hazards regression, we examined 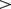
650,000 SNP loci for association with survival. We additionally examined whether the association of significant SNPs with survival was modified by somatic alterations.

**Results:**

Germline polymorphisms at rs4934282 (AGAP11/C10orf116) and rs1857623 (DNAH14) were associated with stage-adjusted survival (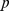 = 1.12e-07 and 1.80e-07, FDR 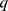 = 1.2e-04 and 2.4e-04, respectively). A third SNP, rs4869 (C10orf116), was additionally identified as significant in the exome sequencing data; it is in near-perfect LD with rs4934282. The associations with survival remained significant when somatic alterations.

**Conclusions:**

Discovery analysis of TCGA data reveals germline genetic variations that may play a role in ovarian cancer survival even among late-stage cases. The significant loci are located near genes previously reported as having a possible relationship to platinum and taxol response. Because the variant alleles at the significant loci are common (frequencies for rs4934282 A/C alleles = 0.54/0.46, respectively; rs1857623 A/G alleles = 0.55/0.45, respectively) and germline variants can be assayed noninvasively, our findings provide potential targets for further exploration as prognostic biomarkers and individualized therapies.

## Introduction

Ovarian cancer accounts for about three percent of all cancers in women and is the fifth leading cause of cancer-related death among women in the United States, with an age-adjusted incidence rate of 12.8 per 100,000 women per year and death rate of 8.6 per 100,000 women per year (2003–2007) [1]. Of the gynecological cancers, ovarian cancer has the highest mortality, with an overall five-year survival rate of 43.7% for white women and 34.9% for black women [1]. The poor survival statistics are attributable to the late stage at which ovarian cancers are diagnosed due to their asymptomatic nature: while stage I tumors have a 92.4% relative survival rate, they account only for 15% of ovarian cancer diagnoses; by contrast, stage III and IV cancers have survival rates of 34% and 18%, respectively, and together account for 65.4% of diagnoses [1]. Response to standard chemotherapy (platinum plus taxane) is highly variable [2], [3], and tends to be poor for advanced cases [2]. Understanding the genetic determinants of ovarian cancer survival and response to treatment may improve these statistics, particularly for stage III and IV patients who comprise the majority of cases. In particular, identifying variations that predict response to chemotherapy allows for the possibility of administering alternate therapies that may improving outcomes.

Previous studies have examined the role of genetic variation in ovarian cancer susceptibility, progression, treatment response, and survival. It has been shown that BRCA1/2 germline mutations contribute to 10–15% of cases [4], and analysis of data from The Cancer Genome Atlas Project (TCGA [5]) has also shown that that BRCA1/2 germline mutation, somatic mutations and promoter methylation effect ovarian cancer survival [5]. Additionally, candidate gene studies have shown that polymorphisms in MDM2, along with TP53 status and SULF1, are associated with ovarian cancer survival [6]–[8]. Recently, Huang and coworkers reported a genetic variation is associated with carboplatin cytotoxicity in vitro and in vivo [3], a finding which may explain differential responsiveness to the standard platinum–based ovarian cancer therapy. The same authors later showed that the identified locus regulates miRNAs that contribute to platinum sensitivity, suggesting a mechanism of action [9].

To date, however, a clinically useful genomic marker of ovarian cancer survival remains elusive. The platinum–associated SNP investigated by Huang was not found to be significantly associated with survival in a validation cohort [3]. Likewise, Bolton and co-workers successfully identified several loci associated with ovarian cancer susceptibility, but those they initially found to be associated with survival failed to reach significance in the validation set [10], although it is hoped that future studies of this cohort will result in established associations with clinical outcome [10]. While tumor gene expression signatures predictive of treatment response and relapse have been reported (e.g., [11], [12]), their clinical utility is limited by the cost, invasiveness, and variability inherent in evaluating tumor gene expression. Likewise, somatic copy number changes in certain genes have recently been reported to influence survival [13], but the utility of measuring CNV as a prognostic test is similarly limited.

The Cancer Genome Atlas Project (TCGA [5]) provides a collection of genomic and clinical data in which associations between genetics and survival can be thoroughly explored. Here, we carry out a genome-wide analysis to systematically investigate associations between *germline* genetic variation and overall survival in TCGA patients diagnosed with ovarian cancer (serous cystadenocarcinoma) [14]. The patients had an age and stage distribution typical of ovarian cancer, as shown in Table 1. Using the clinical and Affymetrix SNP6.0 (“SNP6”) genotype data, we identified two single nucleotide polymorphism (SNP) loci at which the germline genotype is predictive of overall survival in ovarian cancer patients. The associations remain significant after adjusting for stage, and are associated with survival even amongst stage III patients. This suggests that constitutional genetic variation may play a role in treatment response and provides a potential avenue for a non-invasive prognostic biomarker test.

**Table 1:** Stage and age at diagnosis, organized by 5-year survival.

	Censored 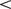 5 yrs	Survival 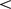 5 yrs	Survival 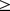 5 yrs	All	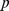
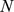	187 (38%)	228 (47%)	74 (15%)	489	
Stage					1.3e-02
I	10	2	2	14	
II	11	6	8	25	
III	140	181	53	374	
IV	26	39	11	76	
Age	58.2 (49.6, 65.5)	59.9 (51.0, 68.1)	62.3 (54.7, 71.4)	59.1 (51.4, 69.1)	6.1e-02

Stage and age at diagnosis of samples, organized by 5-year survival. Median age is reported, with the first and third quartiles given in parentheses. 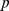 values for the univariate association between stage and survival and age and survival (logrank test) are also given.

## Results

**Figure 1:**
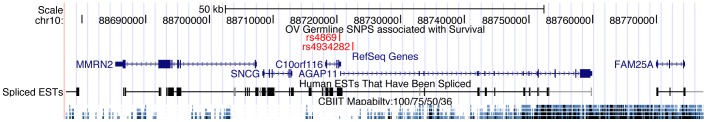
QQ plots. Quantile-quantile plot for observed 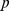 values for the likelihood ratio tests of the stage-adjusted Cox models versus the expected distribution of 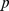 values under independent null hypotheses. Points above the line indicate values that are more significant than expected; a large systematic deviation from this line would be indicative of population substructure driving the results. The two SNPs identified as significant, rs4934282 and rs1857623, lie well above the line and outside the small systematic deviation.

Here, we report the association between germline SNPs and patient survival using TCGA ovarian cancer data. The filtered data comprised a total of 662,521 SNPs assayed in 489 clinically annotated ovarian cancer samples, with stage and age distributions as given in Table 1. Each of the 662,521 SNPs meeting the filtration criteria were tested for association with survival using Cox proportional hazards regression adjusted for stage using a non-additive model. Two SNPs, rs4934282 (A/C) in the gene AGAP11 (previously associated with C10orf116) and rs1857623 (A/G) upstream of DNAH14, showed a statistically significant univariate association with overall ovarian cancer survival, as summarized in Table 2. A 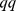 plot of the 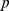-values obtained is given in Figure 1. We additionally computed the per-allele hazard ratios for these SNPs using an additive model, obtaining HR = 0.599 (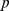 = 1.28e-08) for the 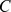 allele at rs4934282 and HR = 1.425 (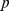 = 1.70e-05) for the 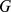 allele at rs1857623. It should be noted that due to the small sample size, the power to detect a SNP with MAF = 0.45 (as these are) with 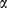 = 1e-06 is 32% for HR = 0.6 and 3.5% for HR = 1.4; it is therefore likely that other SNPs with similar effect sizes may have been missed by chance in this analysis.

**Table 2:** Stage-adjusted survival.

rs4934282 (AGAP11/C10orf116, chr10:88732476)					
	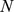 (482)	HR	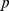	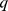	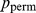
*AA*	146	(ref)	(ref)	(ref)	(ref)
*AC*	231	0.686	8.0e-03	7.5e-01	8.4e-03
*CC*	105	0.355	3.6e-08	3.7e-03	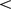 2e-06
Logrank			1.1e-07	1.2e-04	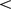 2e-06

Significant survival associations after stratification by stage; rs4934282 and rs1857623 are from SNP6 data, rs4869 is from exome/capture data (29 SNPs tested). All tests of Schoefeld residuals had 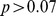, meeting the proportional hazards assumption.

**Figure 2:**
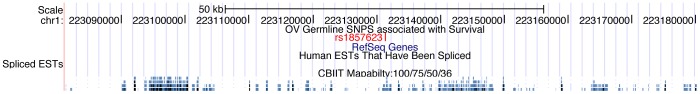
Survival of stage-III ovarian cancer patients by rs4934282 genotype. Kaplan-Meier survival plots for Stage III patients, stratified by germline genotype at rs4934282 (AGAP11): AA, black; AC, blue; CC, red. Confidence intervals are shown as a shaded region around each Kaplan-Meier curve. Censored observations are denoted with vertical ticks. The dashed horizontal and vertical lines mark 50% survival and five years (1825 days) respectively.

**Figure 3:**
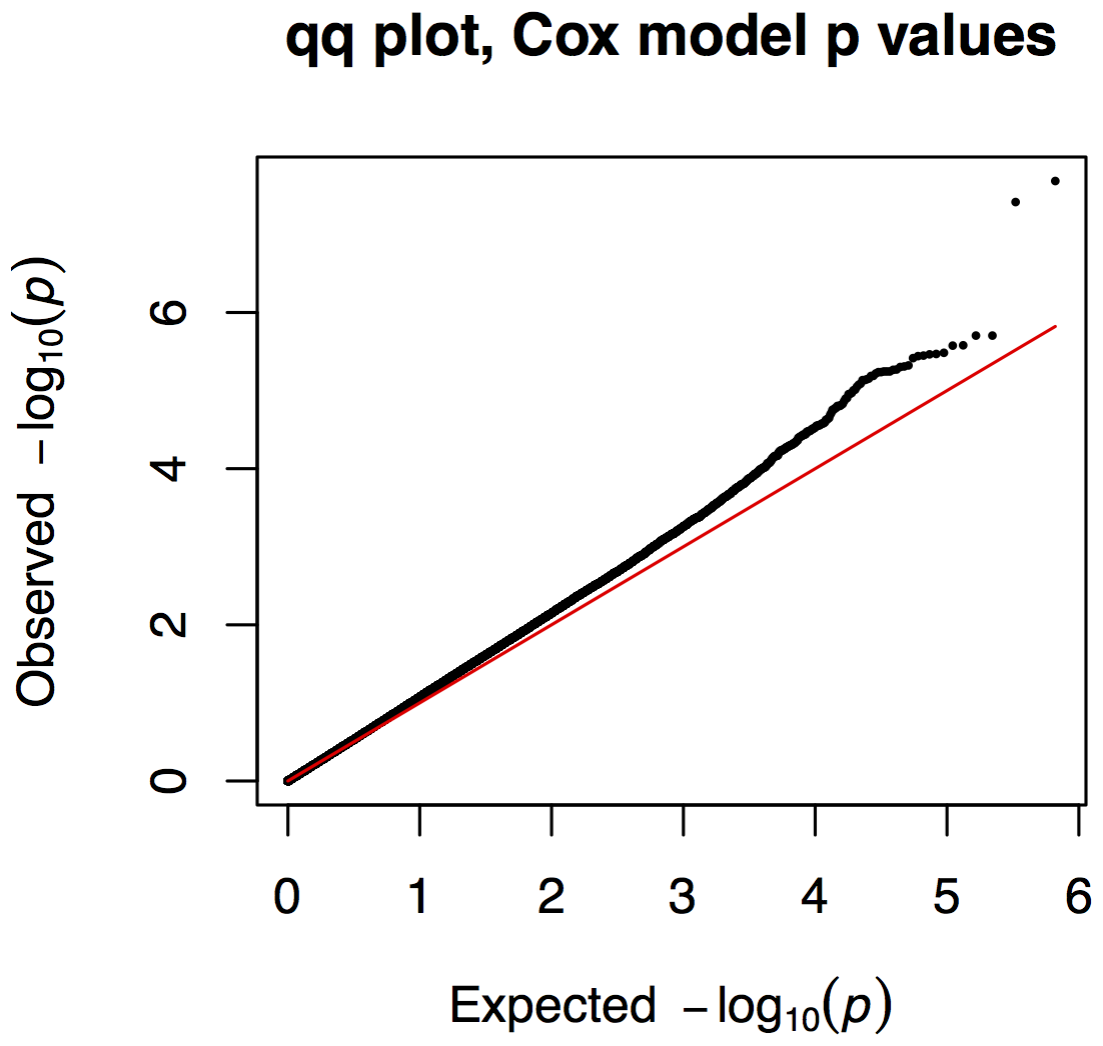
Survival of stage-III ovarian cancer patients by rs1857623 genotype. Kaplan-Meier survival plots for Stage III patients, stratified by germline genotype at rs1857623 (DNAH14): AA, black; AG, blue; GG, red. Confidence intervals are shown as a shaded region around each Kaplan-Meier curve. Censored observations are denoted with vertical ticks. The dashed horizontal and vertical lines mark 50% survival and five years (1825 days) respectively.

To illustrate the effect of rs4934282 (AGAP11/C10orf116) and rs1857623 (DNAH14) germline genotype on survival among patients with similar tumor stage, Kaplan-Meier plots for the 372 Stage III patients are given in Figures 2 and 3. Notably, the CC genotype at rs4934282 in AGAP11/C10orf116 confers a protective effect, nearly doubling the median survival time over the AA genotype group. Additionally, patients with homozygous CC at rs4934282 have a five-year survival rate of 45%, vs. 34% overall for Stage III patients [1].

To further investigate variation in the genomic regions surrounding these SNPs, we examined exome/capture sequencing data (for 375 patients with available germline data) in 100 Kbp windows centered about the two SNPs identified as significant in the SNP6 data, specifically chr10:88672456–88772455 and chr1:223081228–223181227. For ten samples with available whole-genome data, we were able to compare the intronic rs4934282 and rs1857623 Affymetrix SNP6.0 calls to those from the whole-genome sequencing, confirming the validity SNP6 calls. Of the 29 exome/capture SNPs tested (see Table 3) in the 375 samples, only rs4869 in C10orf116 remained significant after adjusting for the multiple hypotheses (FDR 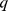 = 9.89e-03). rs4869 is located 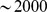
b.p. upstream of rs4934282 and is in near-perfect linkage disequilibrium with rs4934282 (A/C at rs4934282 correlating with C/T at rs4869, respectively). rs4869 encodes a synonymous mutation in C10orf116 (Ile68Ile). We also investigated whether the variant alleles at any of these 29 loci led to deleterious nonsynonymous protein alterations; only five SNPs had mis-sense allelic variations, none of which were predicted to be deleterious (Table 4).

**Table 3:** Cox model results for exome/capture loci.

					Alleles		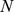 samples				AB		BB			
(lr)6–7 (lr)8–11 (lr)12–13 (lr)14–15 Source	rsID	Chr	Position	Gene	A	B	AA	AB	BB	tot	HR	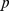 (HR)	HR	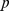 (HR)	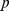 .logRank	
NextGen	rs7074064	10	88673102	BMPR1A	T	C	220	115	29	364	0.94	6.97e-01	1.09	7.35e-01	8.46e-01	
NextGen	rs4447076	10	88686361	MMRN2	A	G	82	81	31	194	1.32	2.12e-01	0.92	8.02e-01	3.59e-01	
NextGen	rs34587013	10	88686602	MMRN2	C	G	287	40	2	329	1.09	7.15e-01			9.33e-01	
NextGen	rs4934281	10	88692330	MMRN2	G	C	1	25	269	295	1.61	6.51e-01	2.15	4.49e-01	4.77e-01	
NextGen	rs10887673	10	88692370	MMRN2	G	A	82	54	10	146	0.70	1.81e-01	0.88	7.96e-01	4.06e-01	
NextGen		10	88694221	MMRN2	G	T	289	20	0	309	1.40	2.86e-01			2.84e-01	
NextGen	rs3750822	10	88695286	MMRN2	T	G	63	21	2	86	0.53	8.97e-02			2.28e-01	
NextGen	rs4244973	10	88707120	MMRN2	T	A	5	31	291	327	1.27	7.08e-01	1.63	4.03e-01	4.45e-01	
NextGen	rs3750823	10	88707134	MMRN2	C	T	139	125	63	327	1.16	3.64e-01	1.07	7.52e-01	6.62e-01	
NextGen	rs1800373	10	88708416	SNCG	A	C	111	115	90	316	1.07	7.02e-01	0.95	8.17e-01	8.34e-01	
NextGen	rs760113	10	88709769	SNCG	C	G	159	72	9	240	0.97	8.73e-01	0.59	2.53e-01	5.13e-01	
NextGen	rs9864	10	88712378	SNCG	A	T	203	105	12	320	0.95	7.47e-01	0.50	1.28e-01	2.98e-01	
NextGen	rs62621086	10	88712453	SNCG	T	G	166	20	2	188	0.76	4.43e-01			4.53e-01	
NextGen	rs2279601	10	88720157	C10orf116	A	G	61	57	42	160	1.45	1.79e-01	1.76	5.66e-02	1.40e-01	
NextGen	rs4869	10	88720292	C10orf116	T	C	100	148	106	354	1.72	4.05e-03	2.06	2.75e-04	7.44e-04	*
NextGen	rs7960	10	88720354	C10orf116	C	T	75	99	38	212	1.23	3.26e-01	1.77	3.65e-02	1.08e-01	
SNP6.0	rs4934282	10	88732476	AGAP11	A	C	138	228	100	466	0.72	2.26e-02	0.37	3.12e-07	1.04e-06	*
NextGen		10	88748032	AGAP11	G	T	252	27	0	279	0.89	7.00e-01			6.99e-01	†
NextGen	rs1240370	10	88748297	AGAP11	T	C	97	118	38	253	1.08	6.85e-01	1.10	7.21e-01	8.99e-01	†
NextGen	rs1240371	10	88748466	AGAP11	C	G	12	26	12	50	2.12	3.22e-01	1.09	9.17e-01	4.62e-01	
NextGen	rs1240407	10	88753935	AGAP11	T	C	16	70	105	191	1.70	2.07e-01	2.17	5.95e-02	1.20e-01	†
NextGen	rs72644240	10	88754336	AGAP11	T	G	3	26	41	70	0.81	7.90e-01	1.01	9.88e-01	8.51e-01	†
NextGen		10	88757859	AGAP11	C	T	20	45	0	65	2.09	9.55e-02			8.85e-02	†
NextGen		10	88757910	AGAP11	G	T	33	34	8	75	1.24	5.75e-01	1.38	5.72e-01	7.87e-01	†
NextGen	rs36104328	10	88758019	AGAP11	A	G	175	58	4	237	0.83	3.92e-01			6.88e-01	†
NextGen	rs2641563	10	88758233	AGAP11	A	G	16	107	161	284	1.70	1.95e-01	2.17	5.26e-02	7.78e-02	
NextGen	rs2641562	10	88758403	AGAP11	A	G	86	119	52	257	1.23	3.08e-01	2.03	2.77e-03	8.99e-03	†
NextGen	rs1745901	10	88758637	AGAP11	C	T	21	124	181	326	1.78	9.58e-02	2.19	2.12e-02	4.38e-02	†
NextGen		1	223127807		C	T	29	25	0	54	1.40	4.69e-01			4.67e-01	
SNP6.0	rs1857623	1	223131228	DNAH14	A	G	145	220	104	469	0.88	3.91e-01	2.04	2.39e-05	1.06e-07	*
NextGen		1	223142168		A	G	19	34	0	53	1.16	7.34e-01			7.34e-01	

Cox model results for exome/capture loci, with significant SNP6.0 loci provided in context. Given are the sample numbers, hazard ratios and associated 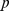 values for each genotype, as well as the logrank 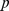 values for the Cox model.

* denotes significant SNPs;

† denotes non-specific regions in the exome/capture data that may reflect variation from another genomic region.

**Table 4:** SIFT and logRE predictions for missense SNPs.

					substitution		SIFT		logRE	
rsID	Chr	Position	Gene	RefSeq	NT	AA	score	prediction	score	prediction
rs3750823	10	88707134	MMRN2	NM_024756	c.G145A	p.G49S	0.15	borderline	0.27	neutral
rs4934281	10	88692330	MMRN2	NM_024756	c.C2191G	p.H731D	0.62	neutral	NA	NA
rs34587013	10	88686602	MMRN2	NM_024756	c.G2728C	p.V910L	0.91	neutral	NA	NA
rs9864	10	88712378	SNCG	NM_003087	c.A329T	p.E110V	0.15	borderline	0.36	neutral
rs2641563	10	88758233	AGAP11	NM_133447	c.A244G	p.I82V	1.00	neutral	NA	NA

SIFT and logRE predictions for missense SNPs. Shown are the location, gene, and RefSeq IDs for the SNPs, the nucleotide (NT) and amino acid (AA) substitutions, and the SIFT and logRE scores and predictions. SIFT scores are classified into predictions as follows: 0.00—0.05, probably damaging; 0.051—0.10, possibly damaging; 0.101—0.20, borderline; 0.201—1.00, neutral. logRE scores are classified into predictions as follows: 1—up, probably damaging; 0.7—0.99, possibly damaging; 0.5—0.69, borderline; 0.0—0.49, neutral.

Finally, we used data derived from normal–paired tumor samples to assess whether the strong effect of germline genotype on survival was significantly mediated or moderated by tumor gene expression gain or loss of copy number in the tumor, or by loss of heterozygosity (see File S1) to test the hypothesis that the effect of germline genotype on ovarian cancer survival might be influenced by somatic events. We found no significant association of tumor gene expression, copy number variation, or loss of heterozygosity in these regions with survival (see File S1). Rather, the large effect of germline genotype at the loci on patient survival is independent of these somatic changes, and appears to suggest that constitutional genetic variation in these regions plays a role in treatment response.

## Discussion

Recent studies have demonstrated that common genetic variants are associated with ovarian cancer risk [15], [16]. However, it remains difficult to predict ovarian cancer survival independent of stage; current clinical findings show that tumor response and extreme drug resistance in vitro are not good predictors of ovarian cancer survival [17], [18]. In our study, we comprehensively tested the SNPs assayed in the TCGA SNP6.0 data for association with survival, and additionally analyzed whole-genome and exome/capture SNPs in the genomic regions surrounding the significant SNP6.0 SNPs. We identified three SNPs in two genomic regions that had a statistically–significant association with survival. As shown in Table 2, the hazard ratios for homozygous minor alleles approached or exceeded two-fold in stage-stratified Cox proportional hazard models, and the per-allele effect sizes for these SNPs using a stage-stratified additive genotype model were HR = 0.599 and HR = 1.425 for rs4934282 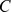 and rs1857623 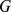, respectively. Interestingly, none of the somatic variations we examined (tumor gene expression, copy number variation, and loss of heterozygosity) were associated either with the germline genotype at these loci or with survival, despite a plausible hypothesis that somatic changes in the tumor might have an effect on the genotype–survival association. Rather, these SNPs are strongly predictive of survival independent of somatic changes that had already occurred in the tumor (see File S1).

**Figure 4:**
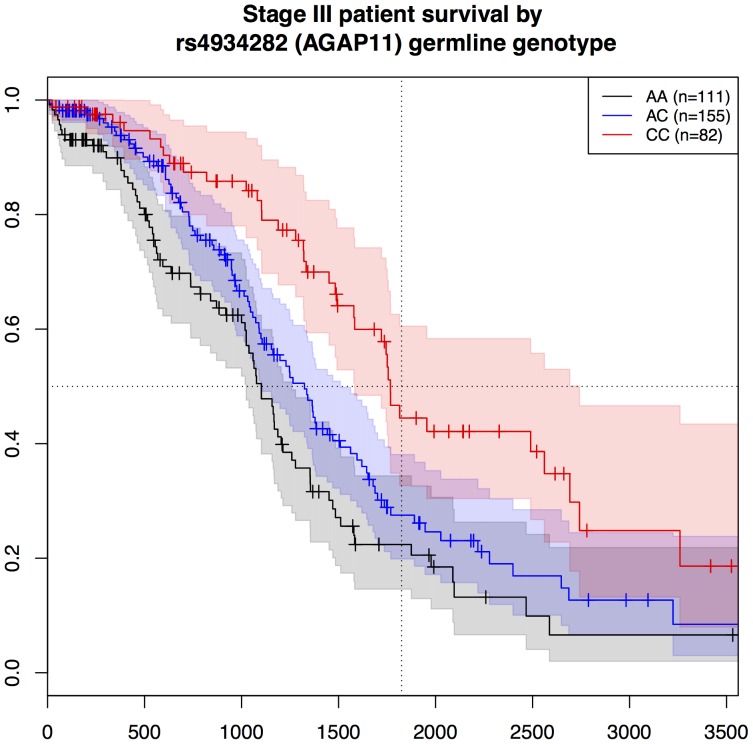
Genomic region containing rs4934282 and rs4869. Detailed description of the genomic region of chromosome 10 containing rs4934282 (second SNP from the right) and rs4869 (shown in green). Note the overlap between AGAP11 and C10orf116.

Two of the survival–associated SNPs are located within a 2200 bp region on chromosome 10 (rs4934282 at chr10:88732476 and rs4869 at chr10:88730312) and are in near–perfect LD in this data. This genomic region is associated with C10orf116 (chr10:88727949–88730672) and AGAP11 (chr10:88730498–88769960), which overlap; the biological significance of the variation probed by rs4934282 and rs4869 may be associated with either. AGAP11 is a member of the ankyrin repeat and GTPase domain Arf GTPase activating protein gene family [19]. C10orf116 (also referred to as APM2) is a protein of unknown function that is homologous to the medium chain of mammalian clathrin-associated protein complex and is involved in vesicular transport in yeast. The genomic region containing rs4934282 and rs4869 is shown in Figure 4.

While little prior evidence exists linking AGAP11 to cancer susceptibility, survival, or treatment response, some evidence exists for the role of C10orf116. C10orf116/APM2 expression has been implicated in other gynecological cancers; for instance, is has been shown to strongly differentiate between the BRCA1 associated breast tumor subclasses ESR1-positive and ESR1-negative [20] and is has been found to be downregulated in utering cancer in a number of studies [21]. More recently, C10orf116 has been shown to exhibit differential expression in different pathological grades of ovarian carcinoma [22] and in the response of breast cancer to chemotherapy [23], [24].

More importantly, there exists from cell lines pointing to C10orf116 as a mediator of cisplatin resistance. Ovarian cancer has been treated with platinum compounds for many years [25], [26], with cisplatin and carboplatin (which has a more acceptible toxicity profile) as a standard therapy for newly–diagnosed stage III ovarian cancers [26], [27]. However, while many patients respond to initial treatment, the five-year survival rates remain poor (34% overall for stage III [1]). APM2 (C10orf1116) has been shown to promote cisplatin resistance when overexpressed in HCT116 cell lines that were sensitive to chemotherapy and radiation [28], suggesting a possible mechanism by which rs4869 and rs4934282 influence survival. Silencing of APM2 by shRNA was shown to enhance the cytotoxic effects of cisplatin on tumor xenografts grown in CD-1 nude mice. Additionally, APM2 was found to be overexpressed in cisplatin resistant gastric cancer cells, but not in gastric cancer cells resistant to 5-FU or doxorubicin [29]. More recently, it was found that rs1649942, a SNP located 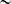
5 Mb upstream of rs4934282/rs4869, had a modest association with carboplatin-induced cytotoxicity and the survival of ovarian cancer patients following carboplatin-based chemotherapy [3]. Although this SNP failed to reach significance in their phase 2 validation analysis (and likewise not significant in our study), it adds to the body of evidence implicating this genomic region in platinum sensitivity.

The third significant SNP, rs1857623, is found in an intergenic region on chromosome 1, 53 Kb upstream of DNAH14 and 136 Kb downstream from CNIH3. DNAH14 belongs to the dynein heavy chain family, a motor protein which attaches to microtubules and walks along cytoskeletal microtubules [30]. The mechanism by which variation in DNAH14 may impact survival is less clear. One possible avenue for future studies is its potential role in the context of taxol therapy: DNAH14 contains the microtubule-binding stalk of dynein motor (pfam12777 at Location:2910–3244 of reference protein NP_001364.1), and it has been demonstrated that taxol binds microtubules [28]. DNAH14 has also been found to be differentially regulated in response to taxane therapy in gastric cancers [31] and doxorubicin therapy in endometrial cells [32].

These findings suggest that consitutional genetic variations in these regions may play a role in ovarian cancer survival even among late-stage cases. However, it should be noted that the results presented here constitute a discovery–based analysis that did not include a validation cohort. As such, the findings may be spurious false positives, and require confirmation in follow–up studies. If validated, these SNPs may have important clinical potential as prognostic biomarkers since germline genotype can be assayed noninvasively and because the variant alleles at the significant loci are common (frequencies for rs4934282 A/C alleles = 0.54/0.46 respectively; rs1857623 A/G alleles = 0.55/0.45, respectvely; both comparable to allele frequencies for the Caucasian CEPH population in HapMap [33]). The significant loci are located in genes previously identified as having a possible relationship to chemotherapeutic response, suggesting that their association with survival may be due to their influence on treatment response. Our study suggests potential targets for prognositic tests and individualized therapies, and provides a basis for follow-up research.

## Materials and Methods

### Data

Data were collected by the TCGA project as described elsewhere [14]. Follow-up times, vital status, tumor stage, and germline genotype data were obtained from the TCGA project [14] via the data portal on 06/03/2011.

#### SNP6 genotypes

Genotype calls for the 906,600 SNP probes assayed using the Affymetrix GenomeWide SNP6.0 platform and processed using Birdseed were obtained from TCGA. Samples that did not pass the TCGA quality control (per the TCGA copy number Sample Data Relationship Format file) were removed. A total of 496 ovarian serous cystadenocarcinoma patients had survival time and germline (either blood or tumor-adjacent normal) genotype data. Genotype calls were coded as 0, 1 or 2 according to the number of variant alleles and filtered according to a Birdseed confidence threshold of 0.05.

The genotype data were subject to additional quality control filtration criteria as follows. SNPs with call rates 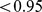 or minor allele frequencies 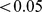 were excluded, as were SNPs out of Hardy Weinberg equilibrium with 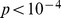. All samples with a call rate below 80% were excluded. Identity by state was computed using the R GenABEL package, and closely related samples with IBS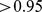 were removed. The SNP and sample filtration criteria were applied iteratively until all samples and SNPs met the stated thresholds. In total, 489 samples and 662,521 SNPs passed were kept in the analysis.

#### Tumor stage

Stage subcategories were coalesced for the purposes of this analysis into summary stage categories yielding four stage classifications (i.e., Stage IA, IB, IC were treated as Stage I, etc.). The number of samples in each stage category is given in Table 1.

#### Exome/capture data

**Figure 5:**
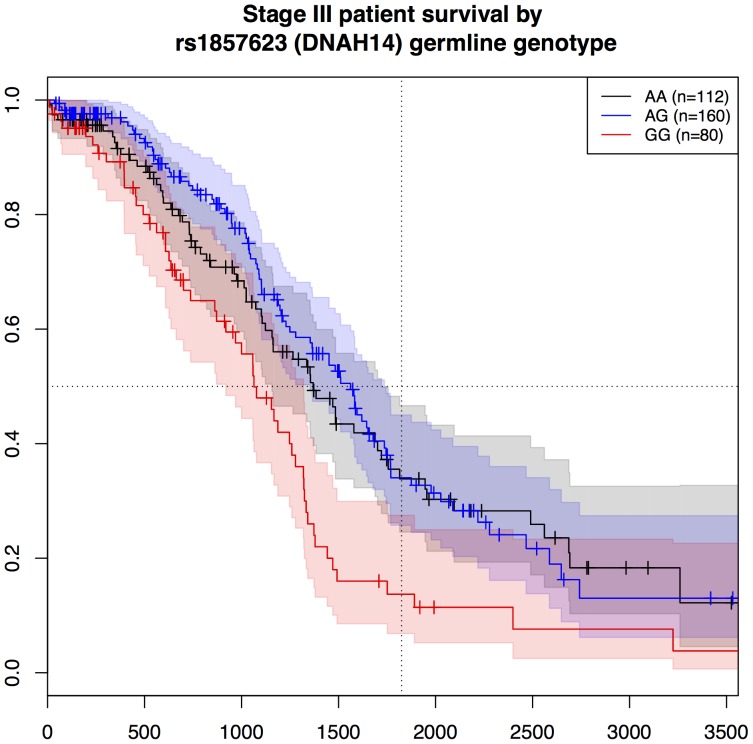
Genomic region surrounding rs4934282. Image from cgwb.nci.nih.gov of selected tracks for genome build NCBI36 (hg18) for the region surrounding two germline variations associated with survival in ovarian cancer in C10orf116/AGAP11 region on chromosome 10. The tracks are a custom track showing the SNPs rs4869 and rs4934282, RefSeq gene, mRNA, spliced ESTs and mapability.

**Figure 6:**
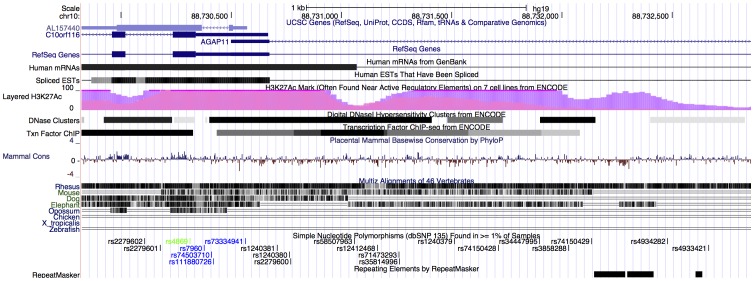
Genomic region surrounding rs1857623. Image from cgwb.nci.nih.gov of selected tracks for genome build NCBI36 (hg18) for the region surrounding a germline variation associated with survival in ovarian cancer upstream of DNAH14 on chromosome 1. The tracks are a custom track showing the SNP rs1857623, RefSeq gene, mRNA, spliced ESTs and mapability.

Next generation exome/capture sequencing data were also retrieved for 375 patients with available germline data. The analysis was restricted to 100 Kbp windows centered about the two SNPs identified as significant in the SNP6 data, specifically chr10:88672456–88772455 and chr1:223081228–223181227. Graphical descriptions of these genomic regions are provided in Figures 5 and 6.

Binary Sequence Alignment/Map (BAM) files were downloaded from dbGAP, using for each sample the largest available normal BAM file. The “mpileup” and “bcftools” features of SAMtools [34] were used to generate the variant call information, with calling criteria as follows: if the coverage in a given sample for a given locus was less than the coverage threshold (see following paragraph), no call was made; otherwise, if non-reference allele frequency was less than 10%, the call was “homozygous reference;” if the non-reference frequency was greater than 90%, the call was “homozygous nonreference;” if it was between 10% and 90%, the call was “heterozygous.”

To set the coverage threshold for the exome/capture data, we compared the exome/capture calls to the SNP6 germline genotype calls for 41 tag SNPs located in those regions. Treating the SNP6 calls as the gold standard for accuracy, we define the “mismatch rate” to be the number of calls for exome/capture and SNP6 data differ, divided by the total number of exome/capture calls made at that coverage depth. As coverage threshold is increased and the exome/capture data becomes more reliable, the mismatch rate decreases, but fewer exome/capture calls can be made. We varied the coverage threshold from 5 to 30, selecting the lowest coverage that yielded a mismatch rate smaller than 0.05. The optimum coverage was 9 (with a mismatch rate of 0.045).

We considered a locus to be informative (ie, having sufficient variation) if at least 20 germline samples had a heterozygous call at that coverage threshold; these criteria yield 29 total informative SNPs in the 100 Kbp regions surrounding rs4934282 and rs1857623, shown in Table 3, which we considered in the analysis.

### Survival analysis

Survival analysis was carried out in R [35] using the “survival” package [36]. For each SNP represented in the data, Cox proportional hazards regression was used to model survival as a function of genotype. Because of the significant association of stage with survival, all models were stratified by stage. Genotype calls were treated as categorical variables with 0 as the referent group to avoid imposing linearity in the number of variant alleles. Each model yielded two hazard ratios per SNP (one for genotype = 1 with respect to genotype = 0 and another for genotype = 2 w.r.t. genotype = 0). The significance of the association was assessed using the logrank (Score) test [37]. A test of Schoenfeld residuals was used to check whether the proportional hazards assumption was met; only models with 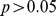 were considered valid. 639,510 SNPs tested met the proportional hazards assumption.

Because the large number of SNPs implies a vast number of hypotheses being tested, multiple testing adjustments were made to the 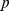 values. This was done in two ways. We report both the false discovery rate [38] (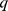) for the 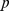 values obtained for the parametric tests described above. In addition, we report permutation 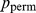 values obtained using 600,000 independent resamplings of the data. Permutation tests, while computationally intensive, are considered the strongest and most appropriate control of type-I error rates in genome-wide studies [39]–[41].

To investigate the existence and effect of any population stratification, the R package GenABEL [42] was used to examine population substructure. The genomic inflation factor was estimated to be 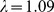, indicating that population substructure, if present, should have no appreciable effect on the results. Using a randomly selected set of 12,000 independent (pairwise LD 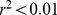) SNPs with MAF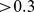, population substructure was examined using principal component analysis. Pairwise plots of the first four components are provided in the File S2. We adjusted the models in two ways: using the first four PCs, and using cluster assignments identified from the PCA using R package mclust [43]. As expected based on 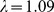, we observed no appreciable changes in the Cox model results (data not shown). The results presented here are therefore not adjusted for population substructure.

### Sequencing data analysis

We compared the SNP6 genotypes at the significant loci (chr10:88722456 and chr1:223131228) to those from whole-genome sequencing data for 10 available samples; all 10 matched the SNP6 calls for the significant SNPs, supporting the SNP6 genotype calls.

For the two SNPs showing significant association with survival in the SNP6 data, we further investigated the surrounding genomic regions using combined whole-genome and exome/capture sequencing data. We investigated 29 SNPs in the the genomic regions surrounding rs4934282 and rs1857623 shown in Table 3 and chosen as described above. Stage-stratified Cox proportional hazards models were then constructed for the germline genotypes as described above. It should be noted neither rs4934282 nor rs1857623 were included due to insufficient exome/capture data (rs4934282 is in an intronic region and hence not assayed in the exome/capture data; rs1857623 had no calls in the majority of samples).

It should be noted that not all the genomic regions contributing to these data have unique sequences. To assess this, we used the “mapability” criteria as implemented in CGWB [44]: for each locus under consideration, we consider a sliding 75 base-pair window containing that locus and attempt to match it to other regions in the genome; the locus is flagged as unique if, for every position of the sliding window, the sequence only maps to the location of the window and no other genomic region. Loci for which some (or all) positions of the sliding window contain sequences that map to multiple genomic regions are flagged with a dagger in Table 3, denoting that the reads contributing to the calls at that locus may be nonspecific.

### Prediction of amino-acid substitutions

We examined the SNPs in Table 3 for mis-sense substitutions using program ANNOVAR [45] and predicted their functional impact on protein sequences with logRE and SIFT. LogRE is the 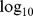 of the ratio of HMMER 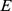-values for the fit to a PFAM motif domain of two amino acid sequences that differ by an amino acid substitution. A logRE score whose absolute value is greater than or equal to 1 indicates that the amino acid alteration is likely to affect protein [46]. SIFT is a sequence homology-based tool that Sorts Intolerant From Tolerant amino acid substitutions and predicts deleterious amino acid substitutions. SIFT values 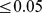 are predicted to be deleterious [47]. Of the SNPs considered above five mis-sense snps were identified: three in MMRN2 (rs3750823, rs4934281, rs34587013), one in SNCG (rs9864), and one in AGAP11 (rs2641563). However, there is no evidence that these amino acid changes have functional impact on the proteins (Table 4).

### Analysis of somatic variations

To test the hypothesis that somatic changes might have an additive or moderating effect on the association between germline genotype and ovarian cancer survival, we used TCGA data derived from paired tumor samples to assess whether tumor gene expression, gain or loss of copy number in the tumor, or loss of heterozygosity were significantly associated with survival. A full description of the methods and results for this analysis is given in the File S1. None of these additional covariates were significant.

## Supporting Information

File S1 **Methods and results of analysis of somatic variations.**
(PDF)

File S2 **Methods and results of population substructure analysis.**
(PDF)

## References

[1] Howlader N, Noone A, Krapcho M, Neyman N, Aminou R, et al.. (2011) SEER cancer statistics review, 1975–2008. Bethesda, MD: National Cancer Institute.

[2] Blackledge G, Lawton F, Redman C, Kelly K (1989) Response of patients in phase ii studies of chemotherapy in ovarian cancer: implications for patient treatment and the design of phase ii trials. British Journal of Cancer 59: 650. 2713253 10.1038/bjc.1989.132 PMC2247161

[3] Huang R, Johnatty S, Gamazon E, Im H, Ziliak D, et al (2011) Platinum sensitivity-related germline polymorphism discovered via a cell-based approach and analysis of its association with outcome in ovarian cancer patients. Clinical Cancer Research 10.1158/1078-0432.CCR-11-0724 PMC3160494 21705454

[4] Pal T, Permuth-Wey J, Betts JA, Krischer JP, Fiorica J, et al (2005) BRCA1 and BRCA2 mutations account for a large proportion of ovarian carcinoma cases. Cancer 104: 2807–2816. 16284991 10.1002/cncr.21536

[5] Bell D, Berchuck A, Birrer M, Chien J, Cramer DW, et al (2011) Integrated genomic analyses of ovarian carcinoma. Nature 474: 609–615. 21720365 10.1038/nature10166 PMC3163504

[6] Han CH, Huang YJ, Lu KH, Liu Z, Mills GB, et al (2011) Polymorphisms in the SULF1 gene are associated with early age of onset and survival of ovarian cancer. J Exp Clin Cancer Res 30: 5. 21214932 10.1186/1756-9966-30-5 PMC3025876

[7] Bartel F, Jung J, Bohnke A, Gradhand E, Zeng K, et al (2008) Both germ line and somatic genetics of the p53 pathway affect ovarian cancer incidence and survival. Clin Cancer Res 14: 89–96. 18172257 10.1158/1078-0432.CCR-07-1192

[8] Marsh S, Paul J, King C, Gifford G, McLeod H, et al (2007) Pharmacogenetic assessment of toxicity and outcome after platinum plus taxane chemotherapy in ovarian cancer: the scottish randomised trial in ovarian cancer. Journal of Clinical Oncology 25: 4528. 17925548 10.1200/JCO.2006.10.4752

[9] Ziliak D, Gamazon ER, Lacroix B, Kyung Im H, Wen Y, et al (2012) Genetic Variation That Predicts Platinum Sensitivity Reveals the Role of miR-193b* in Chemotherapeutic Susceptibility. Mol Cancer Ther 11: 2054–2061. 22752226 10.1158/1535-7163.MCT-12-0221 PMC3438340

[10] Bolton KL, Ganda C, Berchuck A, Pharaoh PD, Gayther SA (2012) Role of common genetic variants in ovarian cancer susceptibility and outcome: progress to date from the Ovarian Cancer Association Consortium (OCAC). J Intern Med 271: 366–378. 22443200 10.1111/j.1365-2796.2011.02509.x

[11] Nikas J, Boylan K, Skubitz A, Low W (2011) Mathematical prognostic biomarker models for treatment response and survival in epithelial ovarian cancer. Cancer Informatics 10: 233–47. 22084564 10.4137/CIN.S8104 PMC3201114

[12] Hartmann L, Lu K, Linette G, Cliby W, Kalli K, et al (2005) Gene expression profiles predict early relapse in ovarian cancer after platinum-paclitaxel chemotherapy. Clinical Cancer Research 11: 2149. 15788660 10.1158/1078-0432.CCR-04-1673

[13] Engler DA, Gupta S, Growdon WB, Drapkin RI, Nitta M, et al (2012) Genome wide DNA copy number analysis of serous type ovarian carcinomas identifies genetic markers predictive of clinical outcome. PLoS ONE 7: e30996. 22355333 10.1371/journal.pone.0030996 PMC3280266

[14] TCGA (2011) The results published here are in whole or part based upon data generated by The Cancer Genome Atlas Pilot Project established by the NCI and NHGRI. Information about TCGA and the investigators and institutions who constitute the TCGA research network can be found at http://cancergenome.nih.gov. (Data accessed 20 July 2011.).

[15] Fasching P, Gayther S, Pearce L, Schildkraut J, Goode E, et al (2009) Role of genetic polymorphisms and ovarian cancer susceptibility. Molecular Oncology 3: 171–181. 19383379 10.1016/j.molonc.2009.01.008 PMC5527888

[16] Bolton KL, Tyrer J, Song H, Ramus SJ, Notaridou M, et al (2010) Common variants at 19p13 are associated with susceptibility to ovarian cancer. Nat Genet 42: 880–884. 20852633 10.1038/ng.666 PMC3125495

[17] Matsuo K, Bond V, Im D, Rosenshein N (2010) Prediction of chemotherapy response with platinum and taxane in the advanced stage of ovarian and uterine carcinosarcoma: a clinical implication of in vitro drug resistance assay. American Journal of Clinical Oncology 33: 358. 19875949 10.1097/COC.0b013e3181af30d3

[18] Rose P, Tian C, Bookman M (2010) Assessment of tumor response as a surrogate endpoint of survival in recurrent/platinum-resistant ovarian carcinoma: A gynecologic oncology group study. Gynecologic Oncology 117: 324–329. 20185168 10.1016/j.ygyno.2010.01.040

[19] Nie Z, Hirsch DS, Randazzo PA (2003) Arf and its many interactors. Curr Opin Cell Biol 15: 396–404. 12892779 10.1016/s0955-0674(03)00071-1

[20] Fernández-Ramires R, Solé X, De Cecco L, Llort G, Cazorla A, et al (2009) Gene expression profiling integrated into network modelling reveals heterogeneity in the mechanisms of brca1 tumorigenesis. British Journal of Cancer 101: 1469–1480. 19826428 10.1038/sj.bjc.6605275 PMC2768459

[21] Arslan AA, Gold LI, Mittal K, Suen TC, Belitskaya-Levy I, et al (2005) Gene expression studies provide clues to the pathogenesis of uterine leiomyoma: new evidence and a systematic review. Hum Reprod 20: 852–863. 15705628 10.1093/humrep/deh698

[22] Skubitz AP, Pambuccian SE, Argenta PA, Skubitz KM (2006) Differential gene expression identifies subgroups of ovarian carcinoma. Transl Res 148: 223–248. 17145569 10.1016/j.trsl.2006.06.001

[23] Sano H, Wada S, Eguchi H, Osaki A, Saeki T, et al (2012) Quantitative prediction of tumor response to neoadjuvant chemotherapy in breast cancer: novel marker genes and prediction model using the expression levels. Breast Cancer 19: 37–45. 21437666 10.1007/s12282-011-0263-8

[24] Hernandez-Vargas H, Rodriguez-Pinilla SM, Julian-Tendero M, Sanchez-Rovira P, Cuevas C, et al (2007) Gene expression profiling of breast cancer cells in response to gemcitabine: NF-kappaB pathway activation as a potential mechanism of resistance. Breast Cancer Res Treat 102: 157–172. 17039268 10.1007/s10549-006-9322-9

[25] Prestayko A, d'Aoust J, Issell B, Crooke S (1979) Cisplatin (cis-diamminedichloroplatinum II). Cancer Treatment Reviews 6: 17–39. 378370 10.1016/s0305-7372(79)80057-2

[26] Jakupec M, Galanski M, Keppler B (2003) Tumour-inhibiting platinum complexes—state of the art and future perspectives. Ergebnisse der Physiologie, Biologischen Chemie und Experimentellen Pharmakologie 146: 1–54. 10.1007/s10254-002-0001-x 12605304

[27] Wang X (2010) Fresh platinum complexes with promising antitumor activity. Anti-Cancer Agents in Medicinal Chemistry 10: 396–411. 20545618 10.2174/1871520611009050396

[28] Alderson K, Holds JB, Anderson RL (1991) Botulinum-induced alteration of nerve-muscle interactions in the human orbicularis oculi following treatment for blepharospasm. Neurology 41: 1800–1805. 1944912 10.1212/wnl.41.11.1800

[29] Kang H, Kim I, Park J, Shin Y, Ku J, et al (2004) Identification of genes with differential expression in acquired drug-resistant gastric cancer cells using high-density oligonucleotide microarrays. Clinical Cancer Research 10: 272. 14734480 10.1158/1078-0432.ccr-1025-3

[30] Pazour G, Agrin N, Walker B, Witman G (2006) Identification of predicted human outer dynein arm genes: candidates for primary ciliary dyskinesia genes. Journal of Medical Genetics 43: 62. 15937072 10.1136/jmg.2005.033001 PMC2593024

[31] Chang H, Rha SY, Jeung HC, Jung JJ, Kim TS, et al (2010) Identification of genes related to a synergistic effect of taxane and suberoylanilide hydroxamic acid combination treatment in gastric cancer cells. J Cancer Res Clin Oncol 136: 1901–1913. 20217129 10.1007/s00432-010-0849-0 PMC11827961

[32] Indermaur MD, Xiong Y, Kamath SG, Boren T, Hakam A, et al (2010) Genomic-directed targeted therapy increases endometrial cancer cell sensitivity to doxorubicin. Am J Obstet Gynecol 203: 1–40. 20444440 10.1016/j.ajog.2010.02.003

[33] The International HapMap Consortium (2003) The International HapMap Project. Nature 426: 789–796. 14685227 10.1038/nature02168

[34] Li H, Handsaker B, Wysoker A, Fennell T, Ruan J, et al (2009) The Sequence Alignment/Map format and SAMtools. Bioinformatics 25: 2078–2079. 19505943 10.1093/bioinformatics/btp352 PMC2723002

[35] R Development Core Team (2011) R: A Language and Environment for Statistical Computing. R Foundation for Statistical Computing, Vienna, Austria. ISBN 3-900051-07-0.

[36] Therneau T, Lumley T (2011) survival: Survival analysis, including penalised likelihood. R package version 2.36-9.

[37] Harrington D, Fleming T (1982) A class of rank test procedures for censored survival data. Biometrika 69: 553.

[38] Benjamini Y, Hochberg Y (1995) Controlling the false discovery rate: a practical and powerful approach to multiple testing. Journal of the Royal Statistical Society Series B (Methodological) 289–300.

[39] Johnson RC, Nelson GW, Troyer JL, Lautenberger JA, Kessing BD, et al (2010) Accounting for multiple comparisons in a genome-wide association study (GWAS). BMC Genomics 11: 724. 21176216 10.1186/1471-2164-11-724 PMC3023815

[40] Cantor R, Lange K, Sinsheimer J (2010) Prioritizing gwas results: A review of statistical methods and recommendations for their application. The American Journal of Human Genetics 86: 6–22. 20074509 10.1016/j.ajhg.2009.11.017 PMC2801749

[41] Han B, Kang H, Eskin E (2009) Rapid and accurate multiple testing correction and power estimation for millions of correlated markers. PLoS Genetics 5: e1000456. 19381255 10.1371/journal.pgen.1000456 PMC2663787

[42] Aulchenko Y, Ripke S, Isaacs A, Van Duijn C (2007) GenABEL: an R library for genome-wide association analysis. 10.1093/bioinformatics/btm108 17384015

[43] Fraley C, Raftery A (2002) Model-based clustering, discriminant analysis, and density estimation. Journal of the American Statistical Association 97: 611–631.

[44] Zhang J, Finney R, Rowe W, Edmonson M, Yang S, et al (2007) Systematic analysis of genetic alterations in tumors using Cancer Genome WorkBench (CGWB). Genome research 17: 1111. 17525135 10.1101/gr.5963407 PMC1899122

[45] Wang K, Li M, Hakonarson H (2010) ANNOVAR: functional annotation of genetic variants from high-throughput sequencing data. Nucleic Acids Research 38: e164. 20601685 10.1093/nar/gkq603 PMC2938201

[46] Clifford R, Edmonson M, Nguyen C, Buetow K (2004) Large-scale analysis of non-synonymous coding region single nucleotide polymorphisms. Bioinformatics 20: 1006. 14751981 10.1093/bioinformatics/bth029

[47] Ng P, Henikoff S (2003) SIFT: Predicting amino acid changes that affect protein function. Nucleic Acids Research 31: 3812. 12824425 10.1093/nar/gkg509 PMC168916

