# Supplementary material for: Discovery Analysis of TCGA Data Reveals Association between Germline Genotype and Survival in Ovarian Cancer Patients
Source: PLoS One. 2013 Mar 21;8(3):e55037. doi: 10.1371/journal.pone.0055037 (PMC3605427; doi:10.1371/journal.pone.0055037)
Supplement: File S1 — Methods and results of analysis of somatic variations. (PDF) [file pone.0055037.s001.pdf]

Supplementary File S1: Analysis of Somatic Variations

To test the hypothesis that somatic changes might alter the effect of germline genotype on ovarian cancer survival, we used data derived from normal–paired tumor samples to assess whether tumor gene expression, copy number changes, or loss of heterozygosity in the previously-identified genomic regions had an additive or moderating role in the association between germline genotype and ovarian cancer survival. We found no evidence that these somatic changes were significantly associated with survival after accounting for germline genotype and stage. A description of the additional somatic analyses and findings is provided in this supplement.

Tumor gene expression. TCGA Level 3 ovarian tumor gene expression data from assays using the Affymetrix HT_HG-U133A and Agilent 244K custom array platforms were downloaded. The Level 3 processed data contained expression levels for 12042 genes in Affymetrix and 17814 genes in Agilent platform; 385 (Affymetrix) and 388 (Agilent) samples had matching SNP6 data, respectively.

We examined the significant SNPs rs4934282 and rs1857623 for eQTL by testing whether the expression of any gene in a patient's tumor tissue was significantly associated with the genotypes at rs4934282 and rs1857623. The nonparametric Kruskal-Wallis test was used to test whether the expression of gene differed between the three genotype groups; neither of the two SNPs showed germline genotype association with the expression of any gene (data not shown). We were also able to test whether the expression of C10orf116 in tumor (the gene closest to rs4934282 in the expression data) was associated with survival using a stage-stratified Cox model; there was no significant association. DNAH14, the gene closest to rs1857623, was not represented in the expression data.

Loss of heterozygosity. To identify instances of loss of heterozygosity (LOH), exome/capture data from matched tumor tissue was obtained for the 375 patients with with germline exome/capture data. The tumor exome/capture data was subject to the same filtration criteria as the germline data (see Materials and Methods).

Using the tumor genotypes paired to the germline genotypes from the exome/capture data, we tested whether evidence of loss of heterozygosity at 29 loci in the 100Kbp regions surrounding rs4934282 and rs1857623 tended to favor the loss of the "A" allele or the "B" allele using a χ^2 test (see Table S1-1). Loci with significant biases toward the loss of a particular allele amongst samples with LOH were also those with very low minor allele frequencies generally, suggesting that this is a sampling artifact rather than a biological bias.

We also tested whether LOH at these loci was associated with survival by sequentially adding loss of the A/B alleles as additive and multiplicative terms to the stage-stratified survival model. We examined both the p values for the hazard ratios for the additive and multiplicative LOH terms as well as ANOVA for the nested models; both revealed no significant association between LOH and survival (all p values > 0.05).

Results are given in Table S1-1.

Copy number variation. For the two SNPs identified as significant in the SNP6 data, we further examined the association of copy number variation both with germline genotype and with survival. The TCGA Level 3 copy number data was downloaded and thresholded at ± 0.2 (segment means less than -0.2 were considered evidence of deletion; segment means greater than 0.2 were considered evidence of amplification).

We examined whether gain or loss of copy number in the regions containing rs4934282 and rs1857623 was associated with germline genotype using a χ^2 test; there was no significant association. We also tested whether gain or loss of copy number in the regions containing rs4934282 and rs1857623 was associated with survival, by sequentially adding copy number gain/loss as additive and multiplicative terms to the stage-stratified survival model. We examined both the p values for the hazard ratios for the additive and multiplicative CNV terms as well as ANOVA for the nested models; both revealed no significant association between CNV and survival (all p values > 0.05).

Results are given in Table S1-2.

Conclusions We found no significant association of these somatic changes with survival, underscoring the crucial role of constitutional genetic variation in treatment response and ovarian cancer survival. While

the strong effect of germline genetic variation independent of somatic changes suggests the importance of these genomic regions in treatment response, it should also be noted that the tumor-derived TCGA data was obtained from the patients at debulking and prior to platinum therapy; it is conceivable that the type and effect of the somatic changes (for example, a tendency toward loss of the protective allele rather than the risk allele in samples with LOH) may be more pronounced after the tumor has been challenged with chemotherapeutic drugs—a potential avenue for follow-up research when appropriate data becomes available.

Exome/Capture Loci				Alleles		*N* samples			LOH			AB		BB		-A		-B			
rsID	Chr	Position	Gene	A	B	AA	AB	BB	-A	-B	$p.\chi^2$	HR	p(HR)	HR	p(HR)	HR	p(HR)	HR	p(HR)	p.ANOVA	
rs7074064	10	88673102	BMPR1A	T	C	220	115	29	10	11	8.27e-01	0.93	6.88e-01	1.09	7.34e-01	1.27	6.06e-01	0.89	7.91e-01	8.40e-01	
rs4447076	10	88686361	MMRN2	A	G	82	81	31	2	4	4.14e-01	1.27	2.98e-01	0.92	8.02e-01						
rs34587013	10	88686602	MMRN2	C	G	287	40	2	2	7	9.56e-02	0.99	9.61e-01					1.90	2.56e-01	5.61e-01	
rs4934281	10	88692330	MMRN2	G	C	1	25	269	1	1	1.00e+00	1.55	6.76e-01	2.16	4.46e-01						
rs10887673	10	88692370	MMRN2	G	A	82	54	10	0	2	1.57e-01	0.70	2.03e-01	0.88	7.95e-01						
	10	88694221	MMRN2	G	T	289	20	0	0	6	1.43e-02	1.02	9.57e-01					2.42	1.49e-01	1.60e-01	
rs3750822	10	88695286	MMRN2	T	G	63	21	2	0	2	1.57e-01	0.48	6.08e-02								
rs4244973	10	88707120	MMRN2	T	A	5	31	291	8	3	1.32e-01	1.21	7.78e-01	1.63	4.04e-01	1.32	6.22e-01			8.30e-01	
rs3750823	10	88707134	MMRN2	C	T	139	125	63	16	11	3.36e-01	1.27	1.75e-01	1.08	7.31e-01	1.06	8.72e-01	0.46	7.65e-02	1.35e-01	
rs1800373	10	88708416	SNCG	A	C	111	115	90	10	12	6.70e-01	1.11	5.93e-01	0.95	8.19e-01	0.94	8.89e-01	0.80	5.72e-01	8.43e-01	
rs760113	10	88709769	SNCG	C	G	159	72	9	2	13	4.51e-03	0.96	8.51e-01	0.59	2.55e-01			1.52	2.75e-01	3.19e-01	
rs9864	10	88712378	SNCG	A	T	203	105	12	8	11	4.91e-01	0.99	9.35e-01	0.50	1.28e-01	0.69	5.36e-01	0.85	7.34e-01	7.74e-01	
rs62621086	10	88712453	SNCG	T	G	166	20	2	2	4	4.14e-01	0.81	6.01e-01								
rs2279601	10	88720157	C10orf116	A	G	61	57	42	2	7	9.56e-02	1.52	1.47e-01	1.75	5.73e-02			0.73	6.10e-01	8.62e-01	
rs4869	10	88720292	C10orf116	T	C	100	148	106	10	15	3.17e-01	1.73	5.46e-03	2.07	2.64e-04	2.17	7.13e-02	0.73	3.73e-01	1.50e-01	
rs7960	10	88720354	C10orf116	C	T	75	99	38	4	7	3.66e-01	1.26	2.91e-01	1.77	3.60e-01			0.45	2.66e-01	2.60e-01	
	10	88748032	AGAP11	G	T	252	27	0	0	20	7.74e-06	1.18	7.47e-01					0.66	5.06e-01	5.15e-01	†
rs1240370	10	88748297	AGAP11	T	C	97	118	38	12	7	2.51e-01	1.08	7.00e-01	1.10	7.21e-01	1.15	7.44e-01	0.74	6.75e-01	8.53e-01	†
rs1240371	10	88748466	AGAP11	C	G	12	26	12	1	5	1.02e-01	1.77	4.61e-01	1.11	9.03e-01			2.20	2.54e-01	2.92e-01	
rs1240407	10	88753935	AGAP11	T	C	16	70	105	11	5	1.34e-01	1.55	3.14e-01	2.17	6.02e-02	1.92	1.30e-01	0.97	9.60e-01	3.56e-01	†
rs72644240	10	88754336	AGAP11	T	G	3	26	41	2	1	5.64e-01	0.72	6.75e-01	0.96	9.55e-01						†
	10	88757859	AGAP11	C	T	20	45	0	0	2	1.57e-01	2.08	1.01e-01								†
	10	88757910	AGAP11	G	T	33	34	8	1	1	1.00e+00	1.20	6.45e-01	1.40	5.61e-01						†
rs36104328	10	88758019	AGAP11	A	G	175	58	4	6	4	5.27e-01	0.72	1.67e-01			1.99	1.68e-01			2.27e-01	†
rs2641563	10	88758233	AGAP11	A	G	16	107	161	10	11	8.27e-01	1.70	1.99e-01	2.18	5.10e-02	1.69	2.32e-01	0.67	4.03e-01	3.21e-01	
rs2641562	10	88758403	AGAP11	A	G	86	119	52	14	15	8.53e-01	1.16	4.82e-01	2.04	2.54e-03	2.29	4.70e-01	0.88	7.52e-01	1.64e-01	†
rs1745901	10	88758637	AGAP11	C	T	21	124	181	10	14	4.14e-01	1.82	8.88e-02	2.20	2.09e-02	1.58	2.89e-01	0.65	2.86e-01	2.85e-01	†
	1	223127807		C	T	29	25	0	0	1	3.17e-01	1.39	4.83e-01								
	1	223142168		A	G	19	34	0	0	14	1.83e-04	1.20	7.04e-01					0.92	8.68e-01	8.68e-01	

Table S1-1: Association of LOH with genotype and survival. Given are the sample numbers, χ^2 tests of association between LOH and germline genotype, hazard ratios and associated p values for the stage-stratified Cox model of survival using both LOH and germline genotype, ANOVA p-values comparing the combined Cox model to the Cox model for genotype alone. † denotes non-specific regions in the exome/capture data that may reflect variation from another genomic region (see Methods).

					Alleles		N samples			CNV			AB		BB		gain		loss			
Source	rsID	Chr	Position	Gene	A	B	AA	AB	BB	gain	loss	$p.\chi^2$	HR	p(HR)	HR	p(HR)	HR	p(HR)	HR	p(HR)	p.ANOVA	
NextGen	rs7074064	10	88673102	BMPR1A	T	C	220	115	29	40	102	2.85e-01	0.95	7.34e-01	1.16	5.75e-01	1.52	6.47e-02	0.85	3.28e-01	8.23e-02	
NextGen	rs4447076	10	88686361	MMRN2	A	G	82	81	31	40	102	8.16e-01	1.29	2.54e-01	0.94	8.68e-01	1.53	1.78e-01	0.99	9.83e-01	4.16e-01	
NextGen	rs34587013	10	88686602	MMRN2	C	G	287	40	2	40	102	4.13e-01	1.08	7.31e-01			1.52	8.06e-02	0.91	5.80e-01	1.68e-01	
NextGen	rs4934281	10	88692330	MMRN2	G	C	1	25	269	40	102	9.51e-01	1.60	6.53e-01	2.13	4.56e-01	1.50	1.36e-01	0.88	5.18e-01	2.39e-01	
NextGen	rs10887673	10	88692370	MMRN2	G	A	82	54	10	40	102	9.01e-01	0.71	2.04e-01	0.90	8.24e-01	1.07	8.68e-01	0.76	3.64e-01	6.13e-01	
NextGen		10	88694221	MMRN2	G	T	289	20	0	40	102	7.47e-01	1.44	2.48e-01			1.48	1.41e-01	0.93	6.93e-01	3.01e-01	
NextGen	rs3750822	10	88695286	MMRN2	T	G	63	21	2	40	102	7.97e-01	0.56	1.27e-01			3.73	1.09e-02	1.98	7.03e-02	2.93e-02	
NextGen	rs4244973	10	88707120	MMRN2	T	A	5	31	291	40	102	3.39e-01	1.28	6.97e-01	1.59	4.29e-01	1.48	1.06e-01	0.90	5.69e-01	2.03e-01	
NextGen	rs3750823	10	88707134	MMRN2	C	T	139	125	63	40	102	9.93e-01	1.15	4.00e-01	1.06	8.00e-01	1.50	9.15e-02	0.90	5.63e-01	1.80e-01	
NextGen	rs1800373	10	88708416	SNCG	A	C	111	115	90	40	102	4.29e-01	1.06	7.48e-01	0.90	5.98e-01	1.59	6.28e-02	0.84	3.43e-01	8.67e-02	
NextGen	rs760113	10	88709769	SNCG	C	G	159	72	9	40	102	3.26e-01	0.98	9.33e-01	0.63	3.31e-01	1.54	1.42e-01	0.92	6.93e-01	3.09e-01	
NextGen	rs9864	10	88712378	SNCG	A	T	203	105	12	40	102	3.66e-01	0.93	6.72e-01	0.53	1.76e-01	1.48	9.64e-02	0.88	4.66e-01	1.52e-01	
NextGen	rs62621086	10	88712453	SNCG	T	G	166	20	2	40	102	2.15e-01	0.68	2.98e-01			1.66	1.67e-01	0.72	1.84e-01	1.21e-01	
NextGen	rs2279601	10	88720157	C10orf116	A	G	61	57	42	40	102	1.26e-01	1.58	1.31e-01	1.71	7.13e-02	1.14	7.21e-01	0.60	9.99e-02	1.51e-01	
NextGen	rs4869	10	88720292	C10orf116	T	C	100	148	106	40	102	1.75e-01	1.73	4.06e-03	2.11	1.88e-04	1.63	3.42e-02	0.82	2.78e-01	3.62e-01	
NextGen	rs7960	10	88720354	C10orf116	C	T	75	99	38	40	102	3.37e-01	1.20	4.08e-01	1.75	4.36e-02	1.70	7.50e-02	0.71	1.38e-01	3.99e-01	
SNP6.0	rs4934282	10	88732476	AGAP11	A	C	138	228	100	49	132	6.48e-02	0.71	2.21e-02	0.37	4.95e-07	1.37	1.22e-01	0.91	5.40e-01	2.10e-01	
NextGen		10	88748032	AGAP11	G	T	252	27	0	40	102	3.30e-01	0.78	4.47e-01			1.62	7.26e-02	0.95	8.07e-01	1.96e-01	†
NextGen	rs1240370	10	88748297	AGAP11	T	C	97	118	38	40	102	3.18e-01	1.04	8.27e-01	1.08	7.81e-01	1.31	3.86e-01	0.82	3.39e-01	3.56e-01	†
NextGen	rs1240371	10	88748466	AGAP11	C	G	12	26	12	40	102	3.22e-01	1.88	4.13e-01	1.09	9.18e-01	2.09	4.51e-02	0.90	8.35e-01	1.87e-01	
NextGen	rs1240407	10	88753935	AGAP11	T	C	16	70	105	40	102	2.53e-01	1.68	2.22e-01	2.11	7.13e-02	1.24	5.42e-01	0.97	8.80e-01	8.08e-01	†
NextGen	rs72644240	10	88754336	AGAP11	T	G	3	26	41	40	102	4.04e-01	0.59	5.28e-01	0.94	9.33e-01	2.42	1.40e-01	1.18	7.47e-01	3.66e-01	†
NextGen		10	88757859	AGAP11	C	T	20	45	0	40	102	9.04e-01	2.81	4.10e-02			2.65	1.13e-01	1.18	7.33e-01	3.14e-01	†
NextGen		10	88757910	AGAP11	G	T	33	34	8	40	102	7.38e-01	1.41	3.86e-01	1.71	3.71e-01	2.45	1.01e-01	1.28	5.83e-01	2.96e-01	†
NextGen	rs36104328	10	88758019	AGAP11	A	G	175	58	4	40	102	6.80e-02	0.74	1.87e-01			1.69	1.06e-01	0.87	5.09e-01	1.95e-01	†
NextGen	rs2641563	10	88758233	AGAP11	A	G	16	107	161	40	102	1.19e-01	1.61	2.50e-01	2.07	6.91e-02	1.56	8.69e-02	0.94	7.37e-01	2.02e-01	
NextGen	rs2641562	10	88758403	AGAP11	A	G	86	119	52	40	102	6.46e-02	1.22	3.25e-01	2.07	2.56e-03	1.69	6.17e-02	0.93	7.47e-01	1.66e-01	†
NextGen	rs1745901	10	88758637	AGAP11	C	T	21	124	181	40	102	2.59e-01	1.73	1.16e-01	2.12	2.73e-02	1.40	1.71e-01	0.89	5.12e-01	2.69e-01	†
NextGen		1	223127807		C	T	29	25	0	128	18	2.00e-01	1.63	3.32e-01			1.83	2.32e-01	0.89	8.84e-01	4.11e-01	
SNP6.0	rs1857623	1	223131228	DNAH14	A	G	145	220	104	173	25	9.71e-01	0.87	3.73e-01	2.03	2.79e-05	1.15	2.98e-01	1.01	9.58e-01	5.78e-01	
NextGen		1	223142168		A	G	19	34	0	128	18	6.34e-01	0.91	8.42e-01			1.42	4.42e-03	2.77	1.67e-01	1.15e-01	

Table S1-2: Association of CNV with genotype and survival. Given are the sample numbers, χ^2 tests of association between CNV and germline genotype, hazard ratios and associated p values for the stage-stratified Cox model of survival using both CNV and germline genotype, and the ANOVA p-values comparing the combined Cox model to the Cox model for genotype alone. † denotes non-specific regions in the exome/capture data that may reflect variation from another genomic region (see Methods).
