# Supplementary material for: Discovery Analysis of TCGA Data Reveals Association between Germline Genotype and Survival in Ovarian Cancer Patients
Source: PLoS One. 2013 Mar 21;8(3):e55037. doi: 10.1371/journal.pone.0055037 (PMC3605427; doi:10.1371/journal.pone.0055037)
Supplement: File S2 — Methods and results of population substructure analysis. (PDF) [file pone.0055037.s002.pdf]

Supplementary File S2: Population Substructure

Of the 489 patients used in this analysis, 431 were white (self-reported), of whom 21 additionally self-reported Ashkenazi descent. (It should be noted that some individuals of the 410 White individuals may also be of Ashkenazi descent, since Ashkenazi ancestry was coded only as "ASHKENAZI" or a missing value, never explicitly non-Ashkenazi.)

Ethnicity was not associated with either genotype at rs4934282 (AGAP11/C10orf116) or rs1857623 (DNAH14), as shown in Table S2-1. In addition, survival was not significantly associated with race, although it was associated with Ashkenazi heritage as shown in Table S2-2. The associations of rs4934282 and rs1857623 with survival were not significantly altered when adjusting for Ashkenazi heritage.

	rs4934282			rs1857623		
	AA	AC	CC	AA	AG	GG
White	130	185	86	124	192	86
Ashkenazi	4	11	6	4	11	6
Black or African American	2	11	9	13	8	2
American Indian or ALASKA Native	0	1	0	0	0	1
Asian	1	11	2	3	7	4
Native Hawaiian or Other Pacific Islander	1	0	0	0	1	0
N/A	6	9	3	6	8	5

Table S2-1: Genotypes by self-reported race and self-reported Ashkenazi descent. There is no association between ethnicity and genotype at either SNP.

	N	H.R.	*p*
White	410	(ref)	(ref)
Ashkenazi	21	0.41	**0.02**
Black or African American	23	1.42	0.17
Asian	14	0.95	0.93
N/A	19	0.60	0.25

Table S2-2: Survival by self-reported ethnicity.

To assess the population stratification, we examined the principal components of the IBS kinship matrix as described in the text. The first 4 PCs contained the bulk of the variance. The R package mclust was used to cluster patients using between 4 and 10 PCs; clustering assignment did not change with the addition of PCs beyond the 4th. In all cases, the best-fit cluster model was obtained with 7 clusters. Cluster assignment and pairwise plots of the first 4 principal components are given in Figure S2-1. As shown in Table S2-3, certain clusters are associated with race/Ashkenazi background: blue, African-American; green, Asian; orange, Ashkenazi Jewish. No cluster exhibited differential survival (including the orange cluster comprising all 20 self-identified Ashkenazi Jews along with 33 Caucasians of unknown ancestry). In addition, no cluster was associated with genotype at either rs4934282 or rs1857623.

	cluster (colors reference Fig S2-1)						
	black	red	green	blue	cyan	magenta	orange
White	44	311	1	1	12	8	33
Ashkenazi	1	0	0	0	0	0	20
Black or African American	0	1	0	20	1	1	0
American Indian or Alaska Native	0	1	0	0	0	0	0
Asian	0	1	8	0	0	5	0
Native Hawaiian or Other Pacific Islander	0	0	1	0	0	0	0
N/A	1	12	1	1	2	2	0

Table S2-3: Ethnic distribution by population cluster.

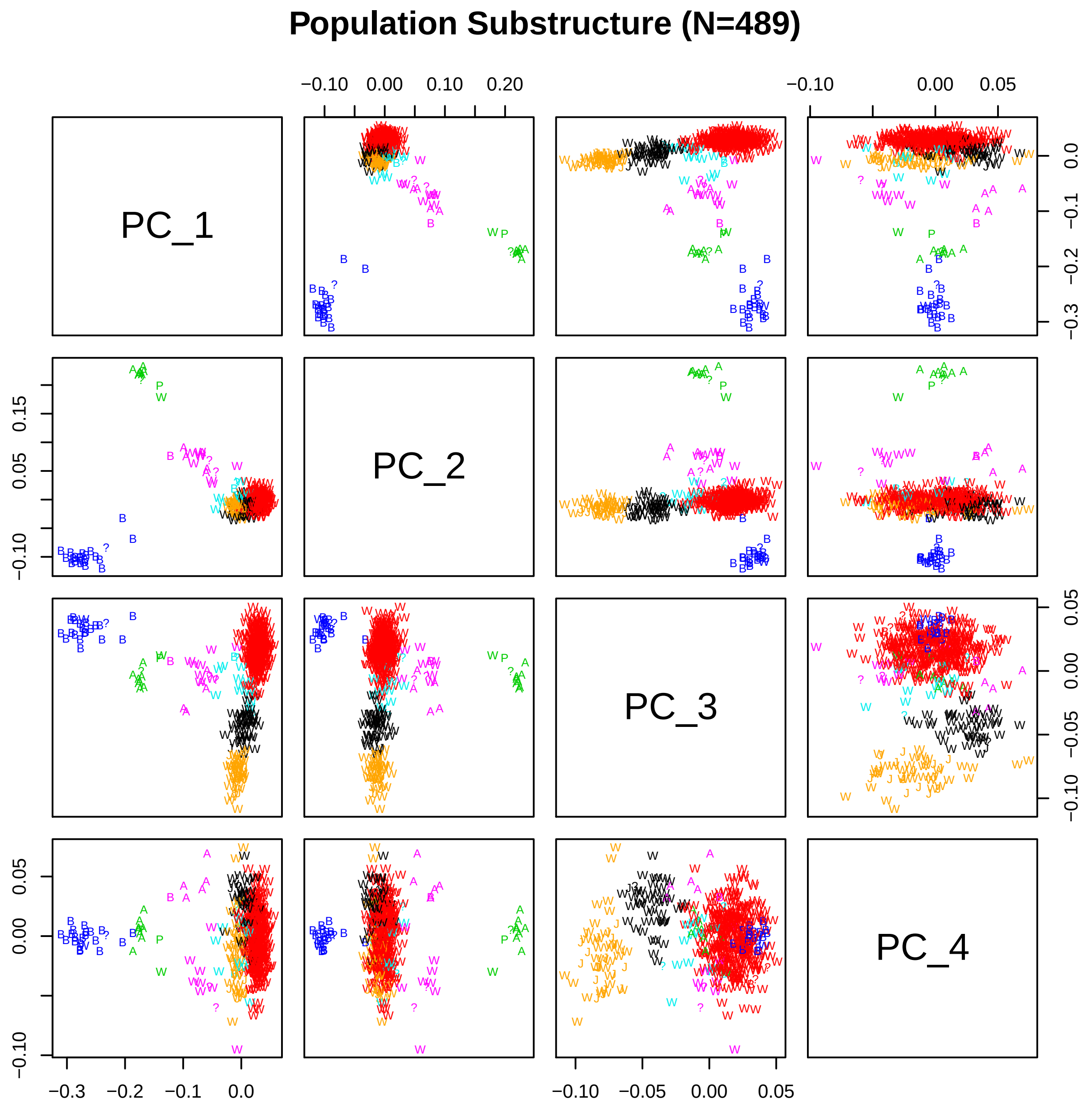

Figure S2-1: Genetic structure of 489 samples based on 12,000 SNPs. Shown are pairwise plots for the principal components of the kinship distance matrix; only the first four components exhibited structure. The principle components were used to cluster the samples using the R package mClust; 7 clusters were obtained, and cluster membership is indicated by color. Self-reported ethnicity is denoted by shape: “W”–white, “B”–Black or African American, “N”–American Indian or Alaskan Native, “A”–Asian, “P”–Native Hawaiian or other Pacific Islander, “J”–Ashkenazi, “?”–unknown.
